# Neutropenic Enterocolitis in a Pediatric Heart Transplant Recipient on Multiple Immunosuppressants

**DOI:** 10.1155/2018/3264921

**Published:** 2018-05-08

**Authors:** Emily E. Miller, Leigh Christopher Reardon

**Affiliations:** UCLA Mattel Children's Hospital, Pediatric Heart Transplant Program, 200 UCLA Medical Plaza Suite 330, Los Angeles, CA 90024, USA

## Abstract

Neutropenic enterocolitis (NE) historically primarily affects pediatric patients with leukemia who are undergoing chemotherapy or who have recently received bone marrow transplants. Although a few case reports have shown NE occurring outside of this typical population, to our knowledge, this is the first published case of NE occurring in the setting of pediatric heart transplant. This patient was diagnosed several months after pediatric heart transplant, with radiographs showing evidence of pneumatosis intestinalis. Although NE does not typically affect solid organ transplant patients, this patient had a variety of risk factors that may have predisposed her to development of NE such as severe neutropenia, supratherapeutic tacrolimus level, immunosuppression with cytotoxic agents, and elevated Epstein-Barr viral load. Fortunately, this patient improved with bowel rest, fluids, antibiotics, and alteration of her immunosuppressive regimen. However, NE can be fatal, and thus it is an important condition to consider, even in patients without leukemia or on chemotherapeutic regimens.

## 1. Introduction

Neutropenic enterocolitis (NE), also known as typhlitis, is a rare and life-threatening condition that is characterized by mucosal injury to the bowel wall in the setting of severe neutropenia. This condition was first reported in patients with leukemia undergoing treatment with cytotoxic induction chemotherapeutic agents, but cases have also been reported in association with aplastic anemia, lymphoma, solid organ cancers, and bone marrow transplants [1]. A recent case report showed development of NE in a pediatric renal transplant [2]. The immunosuppressive agents most commonly implicated in NE include antimetabolites, anthracyclines, and alkylating agents [3]. Incidence in patients with malignancy is unknown, with some studies reporting ranges from 0.8% to 26% [1], though this number is much lower in patients without malignancy.

We present an unusual case of a 10-month-old patient who developed hematochezia following an orthotopic heart transplant, found to have neutropenia, and was subsequently diagnosed with NE. Based on our literature review and to the best of our knowledge, this is the first published case of NE in the setting of heart transplant and a supratherapeutic tacrolimus level.

Informed written consent for the presentation and publication of this case was obtained from the patient's parents.

## 2. Case History

A six-month-old female underwent an ABO incompatible orthotopic heart transplant for idiopathic dilated cardiomyopathy diagnosed at 2 months of age. Her transplant course was unremarkable other than oral aversion, leading to percutaneous endoscopic gastrostomy tube placement. The patient was discharged on typical immunosuppressive therapy including tacrolimus, mycophenolate, and prednisolone without induction therapy per hospital protocol.

**Figure 1:**
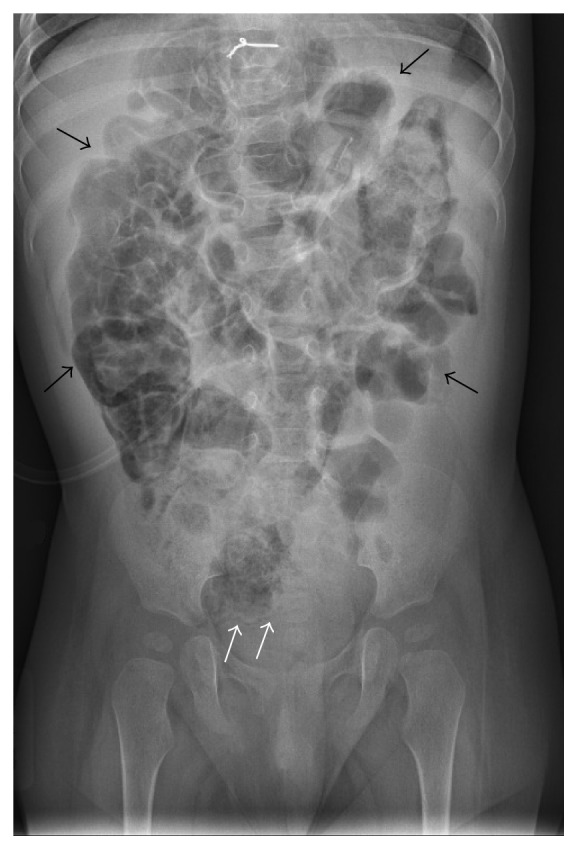
Abdominal X-ray (KUB) showing pneumatosis intestinalis, as evidenced by linear lucencies in the cecum (white arrows) and diffusely edematous bowel (black arrows).

Approximately 2.5 months after transplant, the patient was admitted for multiple episodes of nonbloody, nonbilious emesis. Her workup at that time was reassuring, and she was treated for viral gastroenteritis with rehydration and subsequently discharged home. Shortly after this, the patient's mother noted intermittent pink streaks in the patient's stool, which persisted for 7 weeks and was initially worked out to be from milk protein allergy. The patient then was found to have bright red blood in her diaper and was referred to an outside ER, where she was afebrile and had stable vitals. Workup revealed a normal echocardiogram with no concern for transplant rejection as well as a normal abdominal ultrasound, but abdominal X-ray (KUB) was concerning for diffuse pneumatosis intestinalis due to linear lucencies adjacent to the cecal wall and significantly dilated and edematous bowel (Figure 1). Absolute neutrophil count at that time was 1550 neutrophils/mm^3^, down from 3090 neutrophils/mm^3^ at outpatient clinic appointment 2 weeks prior.

The patient was subsequently transferred to UCLA Mattel Children's Hospital, where a repeat KUB again showed evidence of pneumatosis. Absolute neutrophil count was repeated, with drop to 640 neutrophils/mm^3^ the day of admission and 210 neutrophils/mm^3^ the day after admission. It continued to downtrend for 1.5 weeks after admission with a nadir of 110 neutrophils/mm^3^. Additionally, tacrolimus level was found to be unexpectedly supratherapeutic the day of admission at 28.8 ng/mL (therapeutic range: 8–10 ng/mL). Computed tomography (CT) scan was not obtained, as further imaging was unlikely to change management per discussion with radiology and the providers wanted to avoid additional radiation exposure if possible.

The patient's neutropenia was thought to be secondary to multiple potentially bone marrow suppressing medications, such as mycophenolate, trimethoprim-sulfamethoxazole, and valganciclovir in addition to her supratherapeutic tacrolimus level. These medications were discontinued shortly after admission. Further evaluation of how these medications, particularly mycophenolate, may have contributed to bowel irritation was not pursued given safety concerns with obtaining colonic tissue biopsy in the presence of pneumatosis.

Additionally, rising Epstein-Barr (EBV) viral load may have contributed to immunosuppression, as viral load was 29 at 1.5 months prior to admission, 276 at 2 weeks prior to admission, 289 on the day of admission, and 2471 at a week after admission. The donor was known to be EBV positive while the patient was initially EBV negative pretransplant, hence the reason why EBV viral loads were trended closely before and during hospitalization. The patient received a positron emission tomography-computed tomography (PET-CT) scan after the spike in her EBV viral load due to concern for posttransplant lymphoproliferative disease (PTLD), but this showed no evidence of PTLD and was instead consistent with colitis given concentric wall thickening of the rectosigmoid. Another diagnosis that was initially considered was cytomegalovirus (CMV) colitis, but this was thought to be less likely due to undetected serum CMV level.

The patient was diagnosed with NE shortly after admission based on her neutropenia (particularly on hospital days 2 and 3), pneumatosis on X-ray, and clinical history. She was treated with bowel rest, IV fluids, and IV antibiotic regimen as determined by consulting infectious disease specialists. Initially she received 4 days of piperacillin/tazobactam which was then switched to meropenem for 3 days, but these were discontinued due to concern for myelosuppression. She finished her antibiotic course with 4 days of cefepime and metronidazole. Granulocyte colony stimulating factor (G-CSF) therapy was not given to treat neutropenia per pediatric hematology consultant recommendations. Additionally, pediatric surgery was consulted and did not feel that the patient required surgical intervention; thus no colonic tissue samples were obtained. Stool samples were negative for typical infectious processes.

Four days after admission, repeat KUB showed improvement of pneumatosis and bowel dilation. Followup KUBs were obtained 6 days after admission and 8 days after admission as well, both of which showed resolution of pneumatosis. The patient was then slowly advanced to full PEG tube feeds over the course of a week, which was well-tolerated.

The patient was discharged three weeks after admission after remaining stable on full feeds and with uptrending absolute neutrophil count to 220 neutrophils/mm^3^ at the time of discharge. Her tacrolimus level stabilized during hospitalization with last level of 5.3 on the day of discharge. Additionally, EBV viral load began downtrending as well, with drop to 483 prior to discharge.

At the time of this case report, the patient is stable, with no recurrence of neutropenia or NE following discontinuation of mycophenolate. She remains on tacrolimus and sirolimus for immunosuppression with no concern for transplant rejection.

## 3. Discussion

Immunosuppressive therapy following heart transplant is necessary but can predispose patients to a multitude of infections and complications. Isolated neutropenia after heart transplant occurred in 8% of patients on tacrolimus in one study [4] and has been reported in other cases [5, 6]. However, to our knowledge, this has never led to a reported case of NE in a heart transplant patient.

Other gastrointestinal complications have been reported after cardiothoracic transplants however, with one of the most common findings being pneumatosis, which was the main radiographic finding for our patient as well. Pneumatosis intestinalis, or free air within the bowel wall, is a fairly nonspecific radiographic finding that can correlate with many different disease processes including but not limited to NE. One study showed a 7% rate of pneumatosis over an 8-year period in pediatric patients who received heart and/or lung transplants. None of these patients were diagnosed with NE; however many were found to have rotavirus or Clostridium difficile colitis causing pneumatosis [7]. Interestingly, high tacrolimus level was positively associated with development of pneumatosis in that study [7]; our patient had supratherapeutic tacrolimus level when she was admitted, which suggests the possibility that excess tacrolimus contributed to her clinical presentation.

Another study showed development of pneumatosis in 3% of pediatric heart transplant patients, but all of these patients were asymptomatic and none were diagnosed with NE [8]. The other most common gastrointestinal (GI) complications in this study were cholecystitis, pancreatitis, GI infection with C. difficile and cryptosporidium, and malignancy [8]. One case study reported pneumatosis in a heart transplant patient, with NE as a possible differential diagnosis, but that patient was ultimately diagnosed with intussusception [9].

NE is primarily a clinical diagnosis, as tissue samples from the colon are not usually obtained from surviving patients. There is no universal consensus for diagnostic criteria, but proposed criteria based on a meta-analysis of cases are as follows: major criteria: neutropenia (ANC < 500 × 10^9^ cells/L), bowel wall thickening via CT or ultrasound (>4 mm thickening for ≥30 mm length), fever (>38.3), and abdominal pain [1, 10] with minor criteria: abdominal distension, cramping, diarrhea, and lower GI bleeding [1]. Other studies suggest that plain radiographic findings can support a diagnosis of NE, such as distention of cecum, dilated bowel loops, and pneumatosis intestinalis, as aforementioned [11, 12].

One study suggests that NE should be a diagnosis made histologically [13]; however tissue samples for this purpose are difficult to obtain in living patients. Autopsy findings typically show bowel that is hemorrhagic and necrotic with loss of mucosa and edema of submucosa. Focal ulcerations can be present as well [11, 13].

The exact mechanism and pathophysiology behind NE is unclear, though there are several proposed theories. It is commonly thought that neutropenia leads to reduced mucosal protection, and cytotoxic agents further break down this barrier. This leads to bacterial invasion of the colonic wall, which can thus cause necrosis and perforation. NE almost always involves the cecum, hence sometimes being called “typhlitis,” but involvement can extend to other parts of the colon as well [12].

Treatment for NE is typically bowel rest, antibiotics, fluid support, and total parenteral nutrition as needed [1]. Surgery is not usually necessary, with one study showing that 93% of patients responded to medical management alone [14]. Indications for surgical intervention may be persistent GI bleeding, bowel perforation, and clinical deterioration [1].

The most common risk factors for development of NE are malignancy (particularly leukemia) and treatment with cytotoxic chemotherapeutic agents. A large prospective study of 215 patients with malignancy who had 317 total neutropenic episodes showed that 72.7% of patients with NE were on antimetabolites, 72.7% were on anthracyclines, and 36.4% were on alkylating agents [3].

A few risk factors that may have predisposed our patient to the development of NE were her supratherapeutic tacrolimus level at admission, treatment with an antimetabolite (mycophenolate), treatment with other myelosuppressive medications such as prophylactic antibiotics, and elevated Epstein-Barr viral load. It was unclear if the elevated tacrolimus level contributed to the NE or if the NE altered the tacrolimus absorption. One case report demonstrates NE in a neutropenic patient with infectious mononucleosis, indicating a possible rare association between EBV and NE [15].

Although NE is most often associated with hematologic malignancy, other immunosuppressed and neutropenic patients without malignancy are at risk as well, as is demonstrated in this first reported case of NE in a heart transplant patient. Therefore, this is a diagnosis that should be considered in any neutropenic patient, including those who have recently undergone transplantation, with abdominal pain, fever, diarrhea, bloody stool, and appropriate radiographic findings of bowel thickening, dilation/distention, or pneumatosis.
